# Emergency patients’ satisfaction with humanistic caring and its associated factors in Chinese hospitals: a multi-center cross-sectional study

**DOI:** 10.3389/fpubh.2024.1414032

**Published:** 2024-07-17

**Authors:** Wei Wang, Xinwen Liu, Xiulan Shen, Jichun Zhang, Fengying Zhang, Lulu Liao, Xiaoxiao He, Yilan Liu

**Affiliations:** ^1^Department of Nursing, Union Hospital of Tongji Medical College, Huazhong University of Science and Technology, Wuhan, Hubei, China; ^2^School of Nursing, Huazhong University of Science and Technology, Wuhan, Hubei, China; ^3^Department of Nursing, Wuhan Children’s Hospital, Tongji Medical College, Huazhong University of Science and Technology, Wuhan, Hubei, China; ^4^Department of Emergency Medicine, The First Affiliated Hospital, School of Medicine, Zhejiang University, Hangzhou, China; ^5^Department of Nursing, People’s Hospital of Bortala Mongolian Autonomous Prefecture, Xinjiang, China; ^6^West China School of Medicine, West China Hospital, Sichuan University, Chengdu, China

**Keywords:** humanistic caring, patient satisfaction, emergency service, perception, quality of health care

## Abstract

**Purpose:**

Humanistic caring in clinical practice is important for quality care and patient satisfaction. This study aimed to assess patient satisfaction with humanistic care for emergency patients in China and its associated factors.

**Methods:**

From October 2023 to December 2023, a multi-center cross-sectional survey was conducted across 28 provinces and 87 hospitals in China, using a sampling method for inpatients in emergency department. Patient satisfaction with humanistic care was evaluated by a self-developed questionnaire with 32 items across 6 dimensions. Stepwise multiple linear regression was used to explore associated factors.

**Results:**

A total of 3,003 valid questionnaires were successfully collected, with an effective rate of 86.05%. The emergency patients’ total mean humanistic caring satisfaction score was 4.67 ± 0.66. Age, medical insurance type, specialized emergency department visited, waiting times, whether had accompanied person, hospital level, and hospital type are correlated factors (*P* < 0.05) regarding humanistic caring satisfaction. The correlation analysis showed perceived value, and its three dimensions were moderately correlated with humanistic caring satisfaction. The multiple linear regression showed waiting time (*β* = −0.219, *P* < 0.05), whether had accompanied person (*β* = −0.192, *P* < 0.05), hospital level (*β* = −0.137, *P* < 0.05), functional value (*β* = 0.197, *P* < 0.05), and emotional value (*β* = 0.418, *P* < 0.05) were strong predictors.

**Conclusion:**

Hospitals at all levels should improve patients’ perceived value, shorten waiting times, and provide caregivers with improved humanistic care in the emergency department.

## Introduction

Humanistic caring is considered an ideal that centers around a person’s needs, which highlights patients’ preferences, needs, and expectations for care (1, 2). It is an important aspect of patient satisfaction that helps to improve the quality of medical services and build a harmonious relationship between patients and medical staff (3).

Patient satisfaction is considered the core indicator in medical service evaluation and is also a measure of the quality and effect of humanistic care, often used in studies and clinical settings aimed at providing a reference for the development of humanistic care practice standards (4–6). The patient’s perception of humanistic care attaches great importance to its behavioral intention and includes the likelihood of future utilization and recommendation of services in a particular hospital (7, 8).

Overall patient satisfaction with humanistic care is based on patient experience towards different dimensions in ED (9), including environment, facilities, and patient-medical staff interaction. Emergency patients are involved in multiple departments during their visit, including the pre-screening triage desk, consulting room, examination room, pharmacy, payment office, ward, operating room, etc. Therefore, the measurement of patient satisfaction with humanistic care is affected by many factors and can be complex.

Many studies have explored factors of patient satisfaction, often combining questionnaires with personal information to analyze socio-demographic characteristics associated with satisfaction (4). Age, gender, and marital status have been observed to be strongly related to patient satisfaction (10).

In addition, the chaotic, rushed, and poorly private environment of the emergency room can lead to patient dissatisfaction (11). Additionally, extensive studies have shown that long wait times negatively affect satisfaction scores (12). Strategies to provide humanistic care, such as periodic personal interaction and clinical information during wait time, can increase satisfaction in ED (13). Reversely, patients’ perceptions of their health status and empathy with medical staff were positively linked to patient satisfaction (7).

Patient-perceived value refers to the overall evaluation of the effectiveness of medical technology and services after weighing the perceived efficacy, service, environment, and the cost of time, money, and risk incurred by the patient in obtaining treatment (14). Value-based healthcare emphasizes patient-centered care, giving full consideration to the needs and experiences of patients throughout the entire healthcare process, and committing itself to providing patients with continuous, high-value healthcare services (15). Many studies have also shown that patient-perceived value is an antecedent of patient satisfaction (16). According to the “cognitive-emotional-behavioral” theory (17), when the medical services provided by the emergency department meet the needs of patients and make them feel benefited, the perceived value of patients increases, resulting in the inner emotion of satisfaction, more trust in the hospital during the visit, and then in the behavior of referral and re-consultation. When the patient’s perceived value is low, the patient’s inner needs are not satisfied, and they are more likely to have dissatisfied emotional attitudes. Doctor–patient conflicts and medical disputes are more likely to occur (18).

At present, various patient satisfaction with humanistic care evaluation tools are available to assess patients’ experience and perceptions in ED [e.g., HAMA&HAMD Scale (19), SAS&SDS Scale (20), and CMFS Scale (21)]. However, such scales are mostly subjective in the measurement of psychological and mental status, which ignores the integrity of the emergency process that the patient experienced and has limitations in the characteristics of humanistic care spirit.

Currently, few evaluation models on humanistic caring satisfaction in ED exist. According to literature retrieval, the CAHPS Emergency Department Survey created by Agency for Healthcare Research and Quality (AHRQ) was published in 2014 to measure patient experience in ED, which has a certain influence (22). Consumer Emergency Care Satisfaction (CECSS), developed by Davis in 1988, has been used in many countries and more than 5,000 people have filled it out in several languages. However, these scales have some shortcomings in sample sizes, validity, and applicability (23). In China, tools mostly lack parallel comparison and large data support. Hence, multiple-center cross-sectional survey data on patient satisfaction with humanistic care in ED are lacking at present (6). The objective of the research was to measure patient satisfaction with humanistic care in ED using a self-developed questionnaire: the human caring satisfaction evaluation scale. Specific aims were: (1) to assess overall satisfaction with humanistic care received by ED patients and the practical level and implementation effect of humanistic care in ED and (2) to identify dimensions in ED associated with minor or major patient satisfaction and to analyze the factors associated with humanistic care satisfaction scores to provide a reference for the nationwide evaluation of humanistic care practices in ED.

## Methods

### Ethical consideration

This study was approved by the Ethics Committee of Tongji Medical College of Huazhong University of Science and Technology, with an ethics approval number of 2023-S100. Approval for data collection was obtained from the directors of the institutions that conducted the survey. The eligible subjects were inpatients in ED who volunteered to participate in the study and signed the written informed consent form before the survey. The use of the Perceived Value Scale has obtained the author’s permission and consent. The study complied with the requirements of the Declaration of Helsinki.

### Study design, setting, and sample

From October to December 2023, seven administrative regions of North China, South China, Southwest China, Northwest China, Northeast China, Central China, and East China were selected by convenient sampling method, and 2–4 provinces and cities in the region were selected, among which the ratio of tertiary hospitals to secondary hospitals was selected according to 4:1. All hospitals ranked II and above based on the Chinese hospital categorization system.

The sample size of this study was determined by the following formula: *n* = (Z_α/2_)^2^pq/d^2^, where Z_α/2_ = 1.96 for *α* = 0.05, with an admissible error of 5%, a proportion (P) level of patient satisfaction with humanistic nursing of 63.1%. Then an estimated sample size(n) was 430 after adding a 20% non-response rate.

**Inclusion criteria**:(1) all participants were stable inpatients of the emergency department; (2) all participants or their legal guardians were older than 18 years; (3) patients with good understanding and able to finish the questionnaire independently; and (4) patients with informed consent.

**Exclusion criteria**: (1) Patients with mental illness, cognitive dysfunction, or consciousness disorder who cannot cooperate and (2) patients without smartphones or who cannot answer the questionnaire by smartphone.

### Variables and instruments

**Figure 1:**
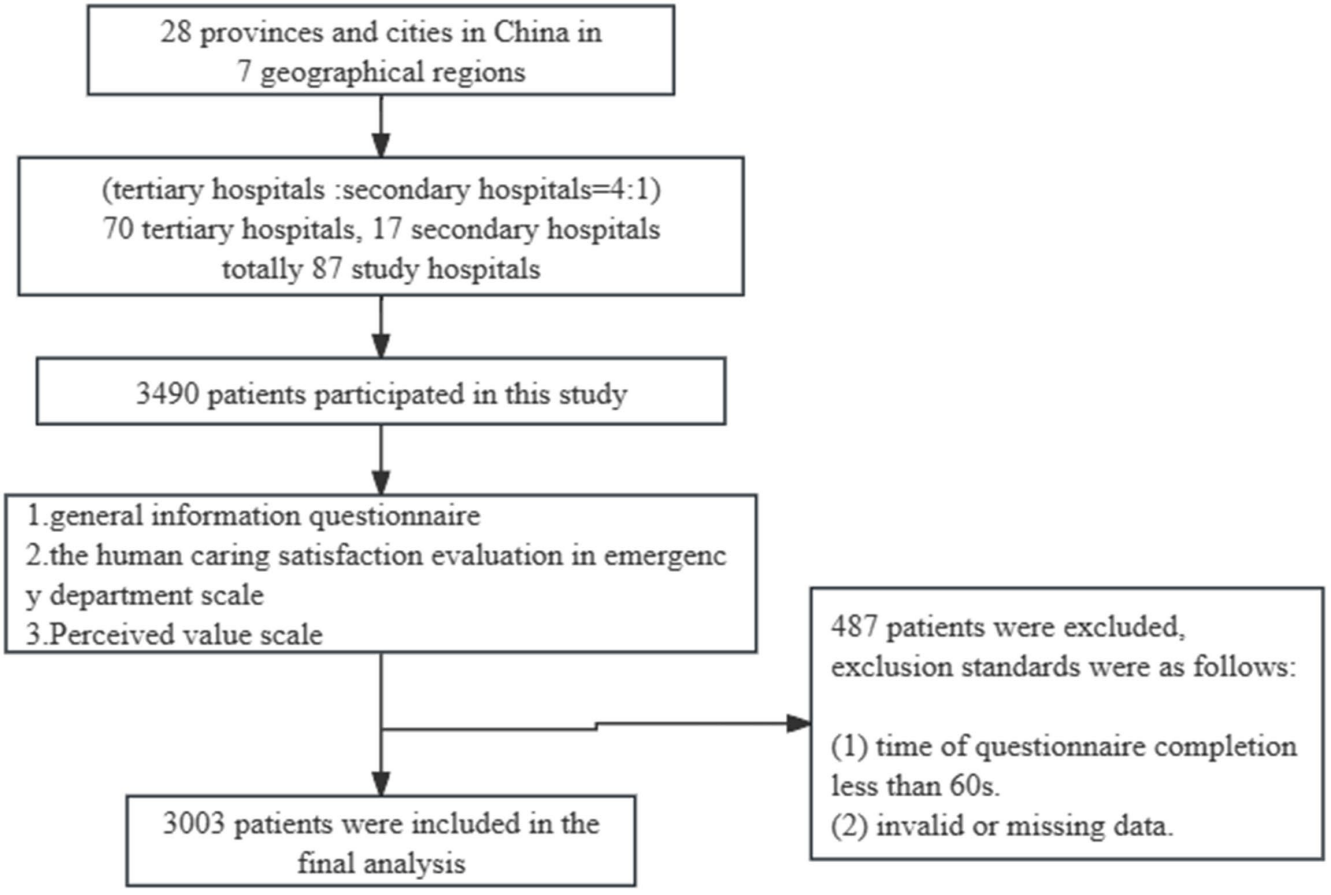
Flowchart of participants throughout the study.

Three questionnaires were used for data collection, including (1) a personal and socio-demographic characteristics questionnaire, (2) a self-developed questionnaire: the human caring satisfaction evaluation in the emergency department scale, and (3) perceived value scale (Figure 1).

#### A personal and socio-demographic characteristics questionnaire

The following data on the socio-demographics and medical condition of each participant were collected: gender, age, education level, marital status, medical insurance type, place of residence, occupation, family monthly income, specialized emergency department (dept) visited, waiting time, whether had accompanied person (AP), hospital level, and hospital type.

#### The human caring satisfaction evaluation scale

The questionnaire’s first draft, created by our research team, was based on the Quality Care Model proposed by Duff (24), which delineates a structure-process-outcome approach. The structural component encompasses patients/families, healthcare providers, and the healthcare system’s characteristics, attributes, and experiences. The process refers primarily to healthcare personnel’s interventions or professional practices, which emphasize care relationships. Elements of care predominantly include joint problem-solving, attentive comfort, respect, encouragement, appreciation of unique meanings, creating a therapeutic environment, fulfilling belonging needs, and addressing basic human needs. Outcomes encompass intermediate and final results, with intermediate results centering on changes in patients’/families’ behaviors, emotions, and cognition during the treatment process, and final results including quality of life and nursing satisfaction. This study applies this model to emergency care in hospitals. The primary participants at the structural level of hospital emergency care include patients, medical personnel, and the emergency department. The attributes of the emergency department include the atmosphere, environmental resources, and facility configuration in the ED. The attributes of medical personnel incorporate professional competence, service attitude, communication skills, etc. Patients’ attributes encompass demographic and social characteristics, as well as individual needs. At the process level, care relationship is paramount, and medical personnel integrates humanistic care into every process of emergency treatment, including precheck, medical consultation, examination, payment, treatment, and transfer. At the outcome level, the effectiveness of humanistic care implementation in hospital emergency departments is evaluated by patient satisfaction. Following consultation with the Chinese Association for Life Care’s Humanistic Care Professional Committee, the questionnaire was used to evaluate the quality of humanistic care in the emergency department. The Delphi method was performed for advice from 18 experts in relevant fields, including emergency experts, nursing experts, medical management experts, and data statisticians. The effective recovery rates for expert consultation were 90.00 and 100.00% in two rounds, respectively. The expert authority coefficients were 0.925 and 0.931, and Kendall’s harmony coefficients were 0.146 (*χ*^2^ = 99.450, *P*<0.05) and 0.152 (*χ*^2^ = 117.550, *P*<0.05). The coefficient of variation in both rounds was at an acceptable level. According to experts’ opinions and suggestions, the questionnaire was revised and finalized by the research group. A total of 436 patients hospitalized in the ED of two tertiary grade A hospitals in Hubei, China, were selected for reliability and validity. Exploratory factor analysis extracted six common factors, and the cumulative variance contribution rate was 88.260%. Confirmatory factor analysis showed that the model was well-fitted, and the factor structure of the scale was stable. The S-CVI of the scale was 0.957, and the I-CVI of each item ranged from 0.824 to 1.000; the overall Cronbach’s α coefficient was 0.968, the split-half reliability was 0.960, and the re-test reliability was 0.876. Overall, the questionnaire’s great reliability and validity are well-supported, which means this self-developed questionnaire has been certified scientifically and practically.

The final questionnaire included 32 items in 6 dimensions: precheck triage caring satisfaction; medical care caring satisfaction; examination, payment, and medicine collection caring satisfaction; treatment, resuscitation, and observation caring satisfaction; transfer caring satisfaction; caring environment and facilities satisfaction, with a total of 32 items. Each item is rated on a 5-point Likert scale (1 = strongly disagree, 5 = strongly agree). The total score ranges from 32 to 160. A higher score indicates greater satisfaction with humanistic care.

#### Perceived value scale

The patient-perceived value was designed by Feng and Duan (16), which was based on the items used by Levesque and McDougall (25) to measure the perceived value of customers and then converted and localized through a scientific approach to validity and reliability. The scale includes three dimensions: functional value, emotional value, and social value, with 10 items in total. Each item scored 1 to 5 points, from “strongly disagree agree” to “strongly agree.” The total score is 50 points. The higher the score, the higher the perceived value of patients. In this study, the overall Cronbach’s α coefficient was 0.985, and the Cronbach’s *α* coefficient of functional, emotional, and social value were 0.968, 0.963, and 0.967, respectively.

### Data collection

The study was conducted by the Questionnaire Star Platform. After obtaining hospitals’ approval for data collection, those questionnaires were distributed by the persons in charge of each hospital, who had been trained by the Chinese Association for Life Care’s Humanistic Care Professional Committee. The questionnaire was completed by inpatients in the emergency department and ED patients transferred to other wards. Patients were asked to carefully read the purpose of the study and precautions before completing the questionnaire. The online questionnaire set all questions as mandatory in order to ensure all items were submitted after completion. Each IP address could only be submitted once, and logic screening was performed to ensure validity. Questionnaires that were submitted within the 60s and had obvious errors such as invalid or missing data were excluded. Two researchers are in charge of data cleaning and verification independently.

### Data analysis

An Excel spreadsheet was exported from the Questionnaire Star platform to establish the original database, and IBM SPSS Statistics for Windows, Version 25.0, was used to analyze the data after excluding invalid questionnaires. Data are described as means with standard deviation (SD) for continuous variables and frequency with percentage for categorical variables. Differences in patient satisfaction with humanistic care according to patients’ characteristics were initially performed through t-test and ANOVA as appropriate, and significant factors with a *p*-value of <0.05 in the univariate methods were analyzed by multiple linear regression to identify factors that are independently associated with patient satisfaction with humanistic care in ED. The overall statistical significance was *P* < 0.05.

## Results

### Demographic characteristics of emergency patients

A total of 3,490 questionnaires were collected from 28 provinces in China. The final analysis included participants from 7 regions: 389(12.95%) from North China, 405(13.49%) from South China, 421(14.02%) from Southwest China, 503(16.75%) from Northwest China, 355(11.82%) from Northeast China, 560(18.5%) from Central China and 370(12.32%) from East China. A total of 87 hospitals were included in the survey, including 70 tertiary hospitals and 17 secondary hospitals. All respondents’ ages range from 18 to 100, with a mean age of 39.43 ± 17.24 years (Table 1).

**Table 1:** Humanistic caring satisfaction by participants’ characteristics (*N* = 3,003).

**Variables**	***N* (%)**	**Score (SD)**	**Statistics**	***P***
**Gender**			**−0.170**	**0.865**
Male	1,269 (42.26)	4.67 ± 0.675		
Female	1,734 (57.74)	4.67 ± 0.651		
**Age (years), M (SD)**	**39.43 ± 17.24**		**3.449**	**<0.05**
18 ~ 45	2,119 (70.56)	4.65 ± 0.715		
46 ~ 69	639 (21.28)	4.73 ± 0.522		
≥70	245 (8.16)	4.71 ± 0.473		
**Education level**			**1.414**	**0.227**
Bachelor degree or above	1,351 (44.99)	4.65 ± 0.706		
College	628 (20.91)	4.68 ± 0.704		
High school/technical secondary school	396 (13.19)	4.66 ± 0.630		
Junior high school	362 (12.05)	4.74 ± 0.508		
Primary school the following	266 (8.86)	4.70 ± 0.538		
**Marital status**			**1.224**	**0.300**
Married	1,986 (66.13)	4.68 ± 0.642		
Single	900 (29.97)	4.64 ± 0.719		
Divorced or separated	61 (2.03)	4.72 ± 0.510		
Widowed	56 (1.86)	4.73 ± 0.510		
**Medical insurance type**			**2.990**	**<0.05**
Town healthcare	875 (29.14)	4.70 ± 0.687		
City healthcare	1,401 (46.65)	4.67 ± 0.634		
New rural cooperative healthcare	330 (10.99)	4.67 ± 0.578		
Commercial insurance	60 (2.00)	4.51 ± 0.756		
Own expense	290 (9.66)	4.63 ± 0.687		
Poverty relief	16 (0.53)	4.79 ± 0.362		
Other	31 (1.03)	4.32 ± 1.273		
**Place of residence**			**0.510**	**0.601**
City	1,994 (66.40)	4.67 ± 0.689		
Towns	462 (15.38)	4.70 ± 0.542		
Rural	547 (18.22)	4.66 ± 0.651		
**Occupation**			**1.664**	**0.092**
Farmer	477 (15.88)	4.70 ± 0.602		
military person	332 (11.06)	4.71 ± 0.626		
Leader	44 (1.47)	4.69 ± 0.593		
employed	749 (24.94)	4.62 ± 0.744		
Worker	286 (9.52)	4.62 ± 0.744		
Self-employed	223 (7.43)	4.70 ± 0.573		
Student	272 (9.06)	4.62 ± 0.707		
freelance	246 (8.19)	4.74 ± 0.558		
Retired	228 (7.59)	4.71 ± 0.576		
Other	146 (4,86)	4.73 ± 0.633		
**Family monthly income (Yuan)**			**1.314**	**0.269**
<3,000	739 (24.61)	4.66 ± 0.649		
3,000 ~ 5,000	1,113 (37.06)	4.70 ± 0.605		
>5,000	1,151 (38.33)	4.66 ± 0.719		
**Specialized emergency department visited**			**2.594**	**<0.05**
Not set	1,484 (49.42)	4.69 ± 0.652		
Emergency surgery	315 (10.49)	4.68 ± 0.666		
Emergency internal medicine	799 (26.61)	4.67 ± 0.612		
Emergency obstetrics and gynecology department	49 (1.63)	4.85 ± 0.729		
Emergency dermatology	74 (2.46)	4.62 ± 0.701		
Emergency E.N.T	25 (0.83)	4.59 ± 1.030		
Other	257 (8.56)	4.54 ± 0.766		
**Waiting time**			**96.760**	**<0.05**
<10 min	1,878 (62.54)	4.78 ± 0.555		
10 min–30 min	729 (24.28)	4.58 ± 0.674		
>30 min	396 (13.19)	4.32 ± 0.907		
**Whether had accompany person**			**7.742**	**<0.05**
Yes	2,442 (81.32)	4.72 ± 0.605		
No	561 (18.68)	4.48 ± 0.838		
**Hospital level**			**2.145**	**<0.05**
Tertiary hospital	2,698 (89.84)	4.68 ± 0.623		
Secondary hospital	305 (10.16)	4.57 ± 0.927		
**Hospital type**			**−3.139**	**<0.05**
General hospital	1929 (64.24)	4.65 ± 0.721		
Specialized hospital	1,074 (35.76)	4.72 ± 0.536		

M = mean; SD = standard deviation.

There were statistical differences in the humanistic caring satisfaction in ED of different patients in terms of age (*F* = 3.449, *P*<0.05), medical insurance type (*F* = 2.990, *P*<0.05), specialized emergency dept. visited (*F* = 2.594, *P* < 0.05), waiting time (*F* = 96.760, *P* < 0.05), whether had AD (*t* = 7.742, *P* < 0.05), hospital level (*t* = 2.145, *P* < 0.05), and hospital type (*t* = −3.139, *P* < 0.05). However, there were no statistical differences regarding gender, educational level, marital status, place of residence, occupation, or family monthly income (*P* > 0.05). Briefly, patients aged 46 ~ 69, patients’ waiting time within 10 min, patients who had an accompanying person, patients in tertiary hospitals, and patients in specialized hospitals reported higher satisfaction with humanistic care in ED (Table 1).

### Humanistic caring satisfaction in ED and its relationship with perceived value

Descriptive statistics are shown in Table 2. The mean score of humanistic caring satisfaction was 4.67 (SD = 0.66), and the average score of perceived value was 4.72 (SD = 0.647). The mean score of dimensions of Humanistic caring satisfaction in ED ranged from 4.61 to 4.71. According to the score of each item, medical care caring satisfaction was higher (4.72 ± 0.680, 4.71 ± 0.700, 4.71 ± 0.696) and transfer caring satisfaction (4.61 ± 0.949, 4.60 ± 0.990, 4.60 ± 0.986) was lower. The top three and the bottom three scores of each item are shown in Table 3.

**Table 2:** Descriptive statistics of scales (*N* = 3,003).

Dimension	Possible range	Total mean ± SD	Average mean ± SD
Total humanistic caring satisfaction	32–160	149.48 ± 21.31	4.67 ± 0.66
Precheck triage caring satisfaction	3–15	14.02 ± 2.407	4.68 ± 0.793
Medical care caring satisfaction	5–25	23.53 ± 3.418	4.71 ± 0.673
Examination, payment, and medicine collection caring satisfaction	6–30	28.19 ± 4.020	4.70 ± 0.659
Treatment, resuscitation and observation caring satisfaction	6–30	27.96 ± 4.605	4.66 ± 0.758
Transfer caring satisfaction	6–30	27.63 ± 5.783	4.61 ± 0.957
Caring environment and facilities satisfaction	6–30	28.09 ± 4.121	4.68 ± 0.676
Perceived value	10–50	47.01 ± 6.22	4.72 ± 0.647
Functional value	3–15	14.08 ± 1.97	4.71 ± 0.685
Emotional value	4–20	18.81 ± 2.50	4.72 ± 0.650
Social value	3–15	14.12 ± 1.87	4.72 ± 0.647

**Table 3:** Ranking of the top three and the bottom three in the humanistic care satisfaction scale for emergency patients (*N* = 3,003).

Items	Dimensions	Average mean ± SD
**The top 3 items**		
Item 4	Medical care caring satisfaction	4.72 ± 0.680
Item 5	Medical care caring satisfaction	4.71 ± 0.700
Item 7	Medical care caring satisfaction	4.71 ± 0.696
**The bottom 3 items**		
Item 21	Transfer caring satisfaction	4.61 ± 0.949
Item 22	Transfer caring satisfaction	4.60 ± 0.990
Item 23	Transfer caring satisfaction	4.60 ± 0.986

(item4:The doctor asked about your condition and asked you about the course of the illness; item5: The doctor clearly informs you of your illness, points out the general diagnosis, and informs you of the examination, treatment plan and risks; item7: Doctors have a good attitude, can listen to your complaints and give you psychological support; item 21: Medical staff take the initiative to help you ask whether there are beds in the ward/clinic to transfer to; item 22: The medical staff shall prepare all the goods and equipment for transshipment, and be ready to deal with emergencies on the way; item 23:Pay attention to your warmth, comfortable posture and privacy protection during transshipment).

Perceived value (*r* = 0.602, *P* < 0.05), functional value (*r* = 0.582, *P* < 0.05), emotional value (*r* = 0.600, *P* < 0.05), and social value (*r* = 0.581, *P* < 0.05) were moderately correlated with humanistic caring satisfaction (Table 4).

**Table 4:** Correlation matrix among variables (*N* = 3,003).

	Variables	1	2	3	4	5
1	Perceived value	1				
2	Functional value	0.966**	1			
3	Emotional value	0.985**	0.921**	1		
4	Social value	0.982**	0.921**	0.962**	1	
5	humanistic caring satisfaction	0.602**	0.582**	0.600**	0.581**	1

**Means *P*<0.05.

### Factors associated with humanistic caring satisfaction in ED

In the multifactor analysis, functional value, and emotional value were taken as independent variables (see Table 5). The humanistic caring satisfaction in ED was considered as the dependent variable. Multiple stepwise linear regression was conducted. Waiting time (*β* = −0.219, *P* < 0.05), AD (*β* = −0.192, *P* < 0.05), and hospital level (*β* = −0.137, *P* < 0.05) were the relevant factors of humanistic caring satisfaction among emergency patients. After controlling these factors, the results showed that functional value (*β* = 0.197, *P* < 0.05) and emotional value (*β* = 0.418, *P* < 0.05) were significant predictors of humanistic caring ability in ED. In total, these six variables explained 40.0% of the variance.

**Table 5:** Multiple stepwise linear regression analysis of humanistic caring satisfaction in ED (*N* = 3,003).

Layered	The independent variables	*B*	SE	*β*	*t*	*P*
First layer	(Constant)	5.380	0.060	—	89.508	<0.05
	Waiting time	−0.219	0.016	−0.237	−13.434	<0.05
	Whether had AD	−0.192	0.030	−0.113	−6.399	<0.05
	Hospital level	−0.137	0.038	−0.062	−3.552	<0.05
Second layer	(Constant)	2.212	0.093	—	23.858	<0.05
	Waiting time	−0.155	0.013	−0.168	−11.686	<0.05
	Whether had AD	−0.114	0.024	−0.067	−4.719	<0.05
	Hospital level	−0.056	0.031	−0.025	−1.793	<0.05
	Functional value	0.197	0.037	0.195	5.372	<0.05
	Emotional value	0.418	0.039	0.392	10.782	<0.05

*B*, unstandardized coefficient; SE, standard error of the unstandardized coefficient; *β*, standardized coefficient; *t*, validity coefficient of the regression.

The first layer, *R*^2^ = 0.077, *F* = 83.934, *P*<0.001; second layer, *R*^2^ = 0.400, △*R*^2^ = 0.323, *F* = 807.357, *P*<0.05.

## Discussion

### Humanistic caring satisfaction in ED is at a high level

The overall humanistic caring satisfaction for emergency patients was high, with a mean score of 4.7, which is in line with the results in similar studies (26). The results have been undeniably improved by the Chinese government’s emphasis on humanistic caring (27), which has developed many regulations and rules to vigorously promote humanistic care in medical institutions. Hospitals at all levels actively responded to the call by setting up humanistic caring wards, organizing staff’s caring ability training, and establishing an evaluation system. Experts in The Humanistic Nursing Professional Committee of China publish consensus and standards for humanistic care in ED. Since then, emergency departments in China have implemented humanistic care according to standards, and patients’ satisfaction has been considerably improved. However, the results may have been biased by the field investigation.

The dimension on the scale listed highest was medical treatment caring satisfaction. A similar study in Iran also showed that the reliability of the healthcare system affected satisfaction in ED (28), which indicates that medical treatment is the core point in the emergency department, with doctors solving patients’ problems and providing them with care and respect.

The lowest level of the scale was transferred caring satisfaction. In our study, transfer refers to intrahospital transport, which means the emergency patients are transferred to specialized departments for follow-up treatment after their condition is relatively stable. Studies have shown that the incidence of intrahospital transport adverse events of emergency patients is 15.2% ~ 34.0% (29), which causes dissatisfaction among patients and even threatens their safety. Previous studies found that drugs, illness severity, instruments and equipment, time, and handover process are risk factors that affect intrahospital transport safety and satisfaction (30–32). Staff who take charge of transfer should communicate with the receiving department, using the standardized communication mode of “identity-situation-background-assessment&action-response&rationale” (ISBAR) to ensure adequate preparation (33). In addition, get all materials and medical equipment ready and checked before transshipment, evaluate the patient’s situation continuously and dynamically during the transportation process, and communicate with the patient, paying attention to privacy protection and warmth.

### Scores of humanistic caring satisfaction in ED about different demographic characteristics

Age, medical insurance type, specialized emergency dept. visited, waiting time, whether had AD, hospital level, and hospital type were associated with differences in humanistic caring satisfaction in ED.

Previous studies confirmed that age can have an impact on patient satisfaction, more specifically, older patients were found to be more satisfied (26). In this study, the age of 46 ~ 69 was found to be more satisfied than the age of 18 ~ 45; however, the age at and above 70 showed less significance about younger age. This could be partially attributed to the less simple size over 70 years old (only accounting for 8.16%), which leads to the insignificant difference. Overall, the results reflected that older patients may have lower requirements than younger patients (3), which could be explained by their learned response to life experience. Another possible contributor to this variance could be the different care received in the emergency department or the experience in the ED, which may have a greater impact on the younger patients’ work and life (34).

As for the type of medical insurance, patients with poverty relief and town healthcare had higher satisfaction for humanistic caring, own expense, and other undefined types, showing the lowest scores. In China, the State Council has released a poverty alleviation plan for the 13th 5-year plan period. To lift all of its poor out of poverty while ensuring adequate health services, the plan develops medical poverty relief to prevent some phenomena of poverty caused by illness (35). As a result, people with medical insurance reported higher satisfaction. When patients are without insurance and have to pay for expensive services in the ED at their own expense, they are under great financial pressure, which makes their satisfaction even lower.

In terms of specialized emergency dept. visited, patients in the emergency obstetrics and gynecology department scored higher, which conflicts with a previous finding that nurses’ humanistic caring ability scored lower in obstetrics and gynecology than other departments. Maternal women, as an important part of emergency patients in obstetrics and gynecology, with higher expectations for services, are more sensitive to the medical environment and humanistic care (36). The reason for our results is not clear right now but it probably has something to do with the fact that there were more patients in other specialized departments (98.37%) than in emergency obstetrics and gynecology (1.63%).

This study adds to the important discussion that hospital with high levels shows better humanistic caring satisfaction, which corresponds with the report that hospital size and type had a significant effect on patient satisfaction scores (37, 38). High-level hospitals can make efforts to improve patients’ perception of emotional aspects. Interventions such as strengthening communication with patients and providing sufficient information about patients’ conditions can improve patient’s trust. Low-level hospitals can strengthen the education and training of employees to improve the diagnosis and treatment level, ensuring patients’ confidence in treatment. In addition, improving the hardware facilities and convenience of medical institutions, as well as creating a humanized environment, may have advantageous effects on patient impressions and satisfaction (39).

Emergency patients with accompanying persons (AD) were found to be strongly associated with humanistic caring satisfaction in ED, which is consistent with the result of the review on the impact of critical illness on relatives (40). Many patients seek treatment in an ED because of sudden illness or life-threatening conditions, physiologically, patients are too weak to participate in daily living, communication, and decision-making, psychologically, patients may experience feelings of depression, anxiety, uncertainty, and even post-traumatic stress disorder. The AP may be a friend, family member, or caregiver, which play an important role in providing emotional support, assisting with daily activities, and facilitating communications and decisions regarding patients’ care. Interventions to address the presence of AP primarily focus on two aspects. On one hand, hospitals make efforts to resolve situations where patients have no AP by providing information for caregivers or support workers. On the other hand, greater attention to emergency care satisfaction should focus on the role of AP (41). The current evidence suggests that medical staff should make efforts to improve AP’s assurance, proximity, information, support, and comfort (42, 43) to have a long-lasting impact on patient’s recovery.

The results of this study showed that ED waiting time was significantly correlated with humanistic caring satisfaction; prolonged waiting times lead to worse patient satisfaction. Many studies have proven the strong impact of waiting time on patient satisfaction (26, 44). In our study, the satisfying waiting time was within 10 min. However, a finding shows a 30-min median waiting time in an emergency care center in central Saudi Arabia (44). The difference in waiting time could be due to the higher number of patients seeking emergency care in China, where it is crucial to minimize waiting times to ensure prompt medical attention for patients.

However, much research showed that it was the perceived but not the actual waiting times that were important to patient satisfaction (45). Furthermore, one study showed that lower ED waiting time had no association with better experience (46), which indicates that the perceived waiting time by patients could be influenced by other factors, leading to potential changes in final satisfaction level. Many efforts have been made to reduce waiting time, adding ED and inpatient beds, and accelerating laboratory and radiology turnaround times, which can improve organizational efficiency (26, 47), while enhancing staff communication skills can promote patients’ expectations and perceived waiting time (13). In addition, many studies observed that patients who were informed of the approximate waiting time showed higher scores for satisfaction (48, 49).

(3) Patient’s perceived value positively related to humanistic caring satisfaction in ED.

In this study, patient’s perceived value was found to be positively correlated with humanistic caring satisfaction in ED (*r* = 0.602, *p* < 0.001). The results of multiple stepwise linear regression analysis indicated that after controlling demographic variables, two dimensions including functional value and emotional value, were the factors predicting humanistic caring satisfaction in ED. Functional value refers to the patient’s rational and economic evaluations, such as the quality or service of the hospital. When patients perceive that the benefits of medical service outweigh the costs they pay, they consider it valuable, and then their satisfaction with humanistic care increases. From this point of view, a well-organized department and the overall quality of humanistic caring services are crucial topics to provide functional value in emergency departments (50). Emotional value refers to the patient’s feelings concerning the service, which predicts the most strongly humanistic caring satisfaction in ED. Studies suggested that communication and information sharing, harmonious nurse–patient relationship, empathy and compassion, respect for patient’s choice, the approachability of doctors, nurses, or staff, and time nurses spend with patients can promote the provision of humanistic care (23, 51). In addition, improving both functional and emotional service encounters for patients in the ED increases perceived value and patient satisfaction and positively influences behavioral intention and loyalty (52).

### Limitation

The possible limitations of this study are related to the methodology used; we only measured emergency patients’ satisfaction with humanistic care during a certain period after intrahospital transfer because of the cross-sectional design, which does not allow causality to be drawn. Furthermore, the distributions of patient’s ages and specialized departments in the ED were not in balance. Besides, all factors only explain 40.1% of the variance, which indicates other factors that contributed to satisfaction should be included.

## Conclusion

The level of humanistic caring satisfaction in the ED was high. It is particularly needed to raise awareness among hospital management about the lack of humanistic caring satisfaction in the intrahospital transport process. Our study suggests that waiting time, accompanying person (AP), hospital level, and perceived functional and emotional value of patients were factors strongly associated with humanistic caring satisfaction in the ED. Hospitals at all levels should make efforts to improve working efficiency to shorten waiting time and provide caregivers with accompany to improve patients’ perceptions and satisfaction in the emergency department. Considering the study’s limitations, future studies should combine longitudinal studies, improve sample diversity, and include more factors to enhance the humanistic care level in ED.

## Data availability statement

The original contributions presented in the study are included in the article/Supplementary material, further inquiries can be directed to the corresponding author.

## Ethics statement

The studies involving humans were approved by the Ethics Committee of Tongji Medical College of Huazhong University of Science and Technology. The studies were conducted in accordance with the local legislation and institutional requirements. The participants provided their written informed consent to participate in this study.

## Author contributions

WW: Conceptualization, Data curation, Formal analysis, Investigation, Methodology, Project administration, Software, Supervision, Validation, Writing – original draft, Writing – review & editing. XL: Investigation, Resources, Writing – review & editing. XS: Investigation, Resources, Writing – review & editing. JZ: Investigation, Resources, Writing – review & editing. FZ: Investigation, Methodology, Writing – review & editing. LL: Formal analysis, Methodology, Supervision, Writing – review & editing. XH: Software, Supervision, Visualization, Writing – review & editing. YL: Conceptualization, Funding acquisition, Resources, Supervision, Writing – review & editing.
